# Understanding flammability and bark thickness in the genus *Pinus* using a phylogenetic approach

**DOI:** 10.1038/s41598-022-11451-x

**Published:** 2022-05-05

**Authors:** J. Morgan Varner, Timothy M. Shearman, Jeffrey M. Kane, Erin M. Banwell, Erik S. Jules, Michael C. Stambaugh

**Affiliations:** 1 Tall Timbers Research Station, 13093 Henry Beadel Drive, Tallahassee, FL 32312 USA; 2 Wildland Fire Laboratory, Department of Forestry and Wildland Resources, Humboldt State University, Arcata, CA 95521 USA; 3 Department of Biological Sciences, Humboldt State University, Arcata, CA 95521 USA; 4 Missouri Tree Ring Lab, University of Missouri, Columbia, MO 65211 USA; 5 Present Address: The Watershed Research and Training Center, Hayfork, CA 96041 USA

**Keywords:** Ecology, Ecology, Environmental sciences

## Abstract

*Pinus* species dominate fire-prone ecosystems throughout the northern hemisphere. Their litter drive fires that control plant community flammability and multiple ecological processes. To better understand the patterns and mechanisms of pine flammability, we measured leaf characteristics (needle length and thickness) and conducted combustion experiments on litter from 31 species. We paired flammability results with bark accumulation data and used phylogenetic generalized least squares regression to examine relationships between physical traits and flammability. Pine flammability varied widely among pines: flame heights and fuel consumption varied three-fold, and flaming and smoldering durations varied three- to six-fold. Subgenus *Pinus* species were the most flammable and subgenus *Strobus* species had the lowest flammability. Needle length was the best predictor of flammability with a significant interaction with subgenus, suggesting that flammability of pines in subgenus *Strobus* was more affected by physical traits than pines in subgenus *Pinus*. Species in the subgenus *Pinus* that accumulated outer bark rapidly also had high flammability, while the relationship was not significant in subgenus *Strobus*. These results highlight the diverse patterns of flammability in North American pines and the complexity in the mechanisms causing differential flammability.

## Introduction

Pines (*Pinus* L.) are a widespread genus of over 100 species distributed throughout the Northern Hemisphere^1,2^. Collectively, these species occur across a range of ecosystems from subarctic, short growing season environments to tropical environments with a year-round growing season^1^. Pines are found in closed canopy forests as well as open savannas and woodlands, and are often the dominant canopy species. This diversity in ecosystems is also reflected in the diversity of forms across pine species, from short statured dwarf pines such as *P. pumila* to tall trees such as *P. lambertiana*^1^. Pines are also linked closely with a wide range of fire regimes^3–5^, which has led to a suite of traits that are hypothesized to be fire adaptations. For example, thick, fire resistant bark^6–8^, “grass stage” seedling physiognomy^9,10^, rapid self-pruning^11,12^, and the “basal crook”^13,14^ are traits that protect vital meristems from the heat of fire. Other traits such as cone serotiny^15,16^ and epicormic resprouting^12,17^ provide a means to quickly re-establish in the post-fire environment.

The flammability of pine litter is an important component of many terrestrial ecosystems and is hypothesized to be a major trait that reinforces the fire regime^18,19^. Litter flammability is a term used to describe the ignition and combustion of surface litter fuels. Flammability has four components—ignitibility (measured time to ignition), sustainability (duration of combustion, effective heat of combustion, heat content, or total heat release), combustibility (mass loss rate, peak heat release rate), and consumability (proportion of fuel consumed by fire)—which can be measured in laboratory or field conditions^20–22^. These metrics translate to fire behavior in the field, characterized by, for instance, rate of spread, fireline intensity, residence time, and fuel consumption^20,22^. While past criticisms of laboratory flammability studies have been made^23^, field evidence from in situ litter fuels in surface fires have corroborated laboratory results^24,25^. All of these fire behavior characteristics can be strong determinants of key demographic rates, such as mortality, growth, and reproduction in pines^3,4^.

The prevalence of highly flammable plants—including their litter—in fire-dependent ecosystems has provoked scientific inquiry and debate about the evolutionary origins of plant flammability for several decades. Mutch^26^ was the first to suggest that fire could be a selective means to increase plant flammability, highlighting that species from fire-prone regions had more flammable litter than species from more fire-naïve regions. Critiques of this early perspective emphasized the lack of an individual fitness benefit from increased flammability^27,28^ or argued that plant flammability could be the by-product of other selective pressures (i.e. exaptation), such as anti-herbivory or drought resistance^29^. More recently, several ‘niche construction’ hypotheses have been developed that provide specific processes that could favor the evolution and spread of a species with increased plant flammability. The “kill thy neighbor” hypothesis poses that flammable traits could be favored in a species that has a pre-existing fitness benefit to fire (e.g., post-fire regeneration) and where fire spreads to less flammable neighbors that lack such a fitness benefit^30^. The “pyrogencity as protection” hypothesis, states that increased plant flammability could confer protective advantages in which lower residence times, and thus, less soil heating, may increase survival of underground storage organs or seeds^31^. A similar case could be made between flammability and other protective traits (e.g., bark thickness) that can increase survivorship during fire.

The protective properties of thick bark in relation to fire have been well-studied^6,32,33^. Bark investment not only differs among species, but changes as individuals age. Species with a negative allometry develop thicker bark at younger ages and reduce relative investment later in life, while species with positive allometry have thin bark at young ages and increase investment later in life^7^. These differences in bark investment are presumed to reflect the evolutionary fire history of a species with early bark investment occurring in species that experience and are able to survive frequent fires^7,12,34,35^. Because saplings are more vulnerable to fire than mature trees, differences in bark thickness between fire-tolerant and fire-intolerant species should be most apparent in sapling stems. Although bark serves functions other than protection from fire^36^, early investment in rhytidome (outer bark) thickness is necessary to survive frequent fire regimes and recruit into the canopy.

**Figure 1:**
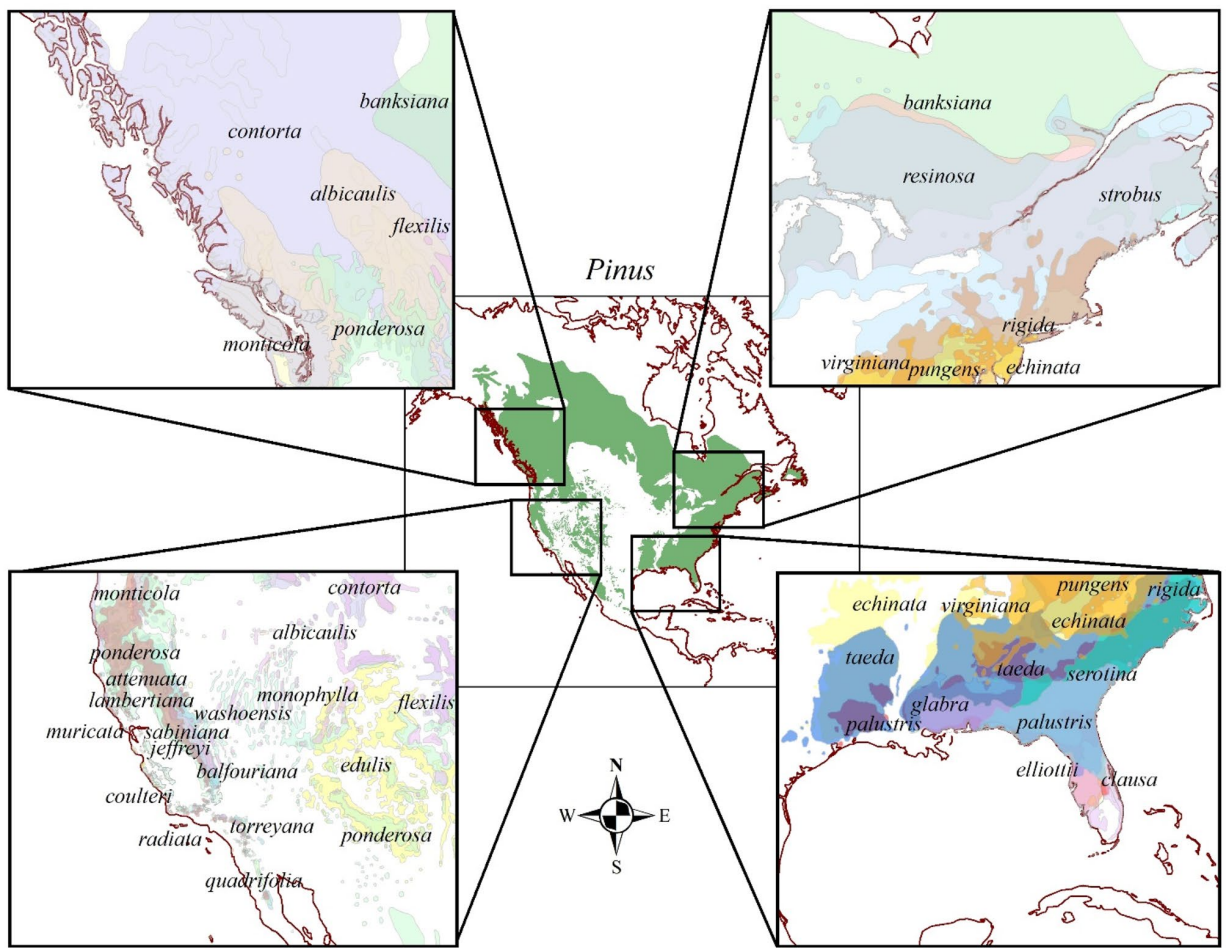
Distribution of North American pine species included in this study. Map generated with ESRI ArcMap version 10.8.1.14362 using species native ranges by Little^58^ and digitized in^59^.

In this study, we focused on 31 pine species collected from the USA (Fig. 1). We asked the following questions: (1) is the variation in pine litter flammability associated with physical traits (needle length and thickness)?; (2) how does litter flammability and its relationship with physical traits vary with phylogeny?; and (3) independent of phylogeny, are there consistent associations between litter flammability and bark thickness among pines? We hypothesized that pines with longer and thinner leaves would be most flammable, based on similar analyses in western USA conifers^35^. We also hypothesized that the two subgenera (*Pinus* and *Strobus*) would differ due to *Pinus* occupying more fire-prone sites than *Strobus*^3,4^. As protective traits vary with fire regime^4,35^, we hypothesized that bark accumulation would be linked to flammability across the genus. Lastly, we sought to compare litter flammability to historic fire frequency, a primary fire regime characteristic that varies widely among pines^3,15^. We hypothesized that species’ range-wide fire frequency would be correlated with both flammability and bark traits. We see these analyses as a necessary step toward a broader understanding of fire-adaptive strategies in pines, an approach that could be expanded to other genera and regions.

## Results

### Patterns of pine flammability across species

Physical traits varied widely across the 31 pine species. Average needle length, thickness, and litter depth ranged from 3.1 to 30.0 cm, 0.25 to 2.00 mm, and 1.9 to 6.0 cm, respectively. Flammability also varied across the pines. Maximum flame height averaged between 20.4 to 87.2 cm, with flame and smolder times ranging from 38.6 to 253.9 s and 213.2 to 801.7 s, respectively. Average fuel consumption varied widely, ranging from 30 to 93% (SI: Appendix 2). The allometric coefficient for bark thickness^7^ for the pine species examined in this study ranged from 0.75 to 1.20 and sapling outer bark thickness ranged from 0.09 to 0.76 cm.

**Figure 2:**
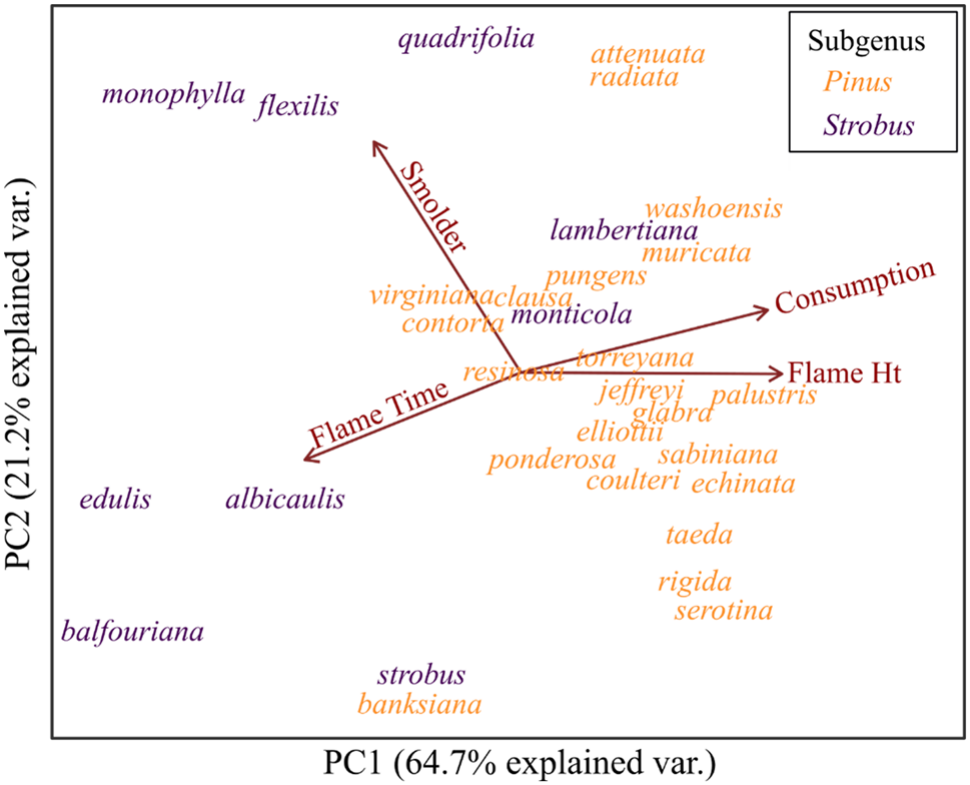
Principal component analysis of the flammability of 31 North American pine species.

Combining the flammability metrics into a PCA resulted in a two-axis solution that explained 85.9% of the variability in the data. The first principal component (PC1) explained 64.7% of the variability and was positively related to flame height and percent fuel consumption and negatively related to flame duration (Fig. 2). The second principal component (PC2) explained an additional 21.2% of the variation and was positively correlated with smoldering duration.

The flammability of the 31 pines was variable and illustrated stark phylogenetic differences. PC1 revealed a suite of species with high flammability (related to flame height and consumption) including the eastern *P. palustris*, *P. echinata*, *P. serotina*, *P. taeda*, and *P. rigida* and western *P. sabiniana*, *P. washoensis*, and *P. muricata* (all members of subgenus *Pinus*; Fig. 2). The low flammability end of PC1 included the western *P. balfouriana*, *P. edulis*, *P. monophyla*, *P. flexilis*, and *P. albicualis* (all members of subgenus *Strobus*). PC2 was also wide-ranging, but differences based on phylogeny were not apparent.

### Drivers of pine litter flammability

**Figure 3:**
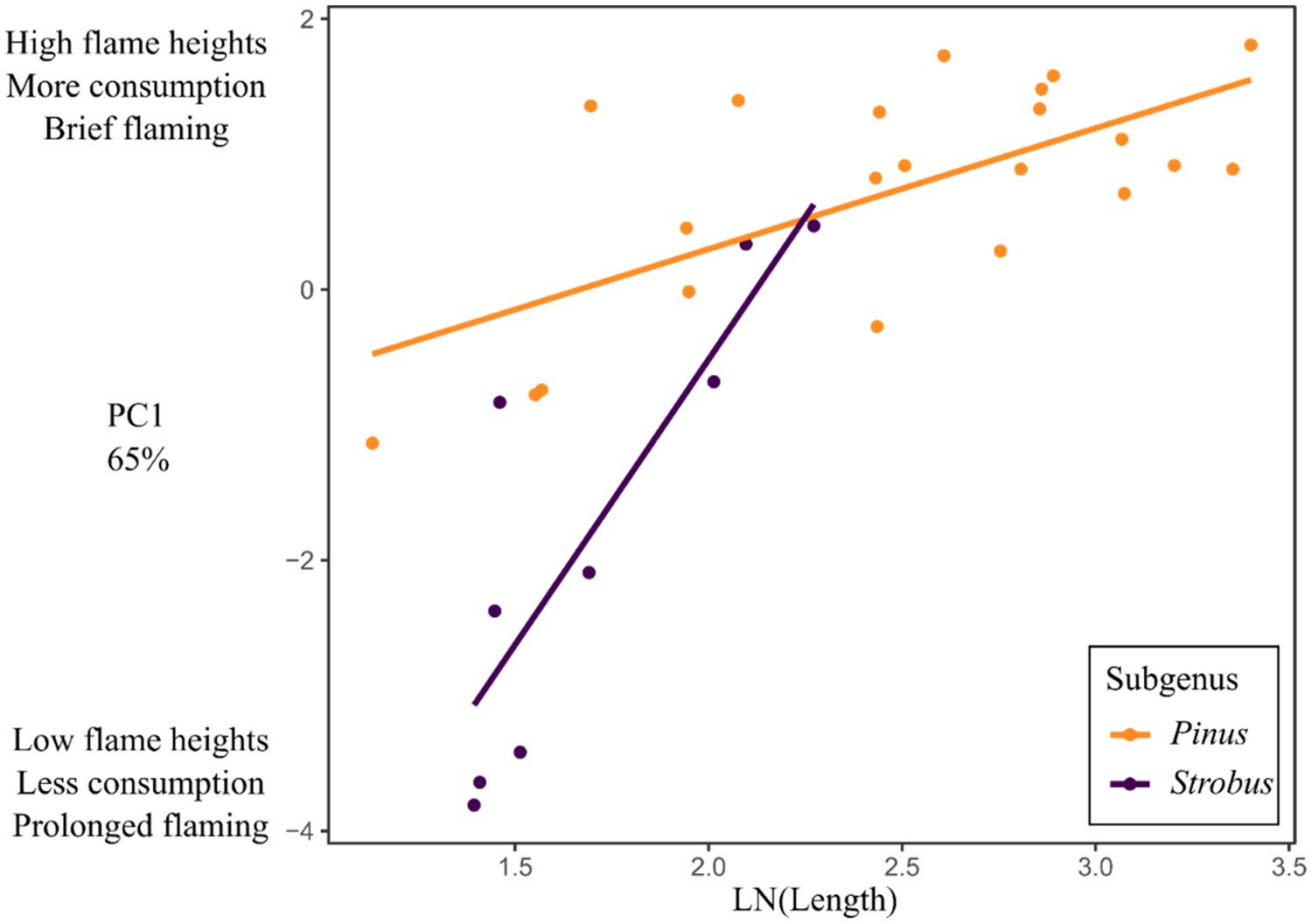
Relationship between pine needle length (log transformed) to the first principal component (PC1) of combined flammability for the 31 North American pine species, segregated by subgenus (*Pinus* and *Strobus*).

Needle length (*P* < 0.0001) (log transformed) and its interaction with subgenus (*p* = 0.0003) was the model that best explained pine litter flammability (*R*^2^ = 0.79, *p* = 0.0003, Table 1). Species with longer needles had greater flammability; this relationship was more pronounced in the *Strobus* subgenus (Fig. 3). There was no significant phylogenetic signal detected in the residual error of the model (λ = 0, 95% CI = 0–0.8). When models were fit to each subgenus separately, however, a phylogenetic signal was found for the *Pinus* subgenus (*R*^2^ = 0.21, *p* = 0.018, λ = 0.75, 95% CI = 0.23–0.98) but not for the *Strobus* subgenus (*R*^2^ = 0.69, *p* = 0.003, λ = 0, 95% CI = 0–0.59).

**Table 1:** Model selection table for flammability of 31 North American *Pinus* species.

Model	*R*^2^	df	Loglik	AICc	Delta	Weight
~ LN(Length) × Subgenus	0.79	4	−32.117	73.8	0	0.872
~ Length × Subgenus	0.50	4	−34.148	77.8	4.06	0.114
~ LN(Depth) × Subgenus	0.32	4	−37.72	85	11.21	0.003
~ LN(Thickness) × Subgenus	0.31	4	−37.86	85.3	11.49	0.003
~ Thickness × Subgenus	0.30	4	−38.055	85.6	11.88	0.002
~ LN(Length)	0.25	2	−40.647	85.7	11.95	0.002
~ LN(Depth)	0.18	2	−41.64	87.7	13.94	0.001
~ Depth × Subgenus	0.23	4	−39.615	88.8	15	0
~ Depth	0.14	2	−42.371	89.2	15.4	0
~ LN(Thickness)	0.14	2	−42.402	89.2	15.46	0
~ Thickness	0.13	2	−42.613	89.7	15.88	0
~ Length	0.10	2	−43.177	90.8	17.01	0

Models were fit using the first principal component of flammability (PC1) as the dependent variable.

Subgenus was either *Pinus* or *Strobus*.

*LN* natural log, *length* needle length, *thickness* needle thickness, *depth* litter depth.

### Linking litter flammability and bark protection

**Figure 4:**
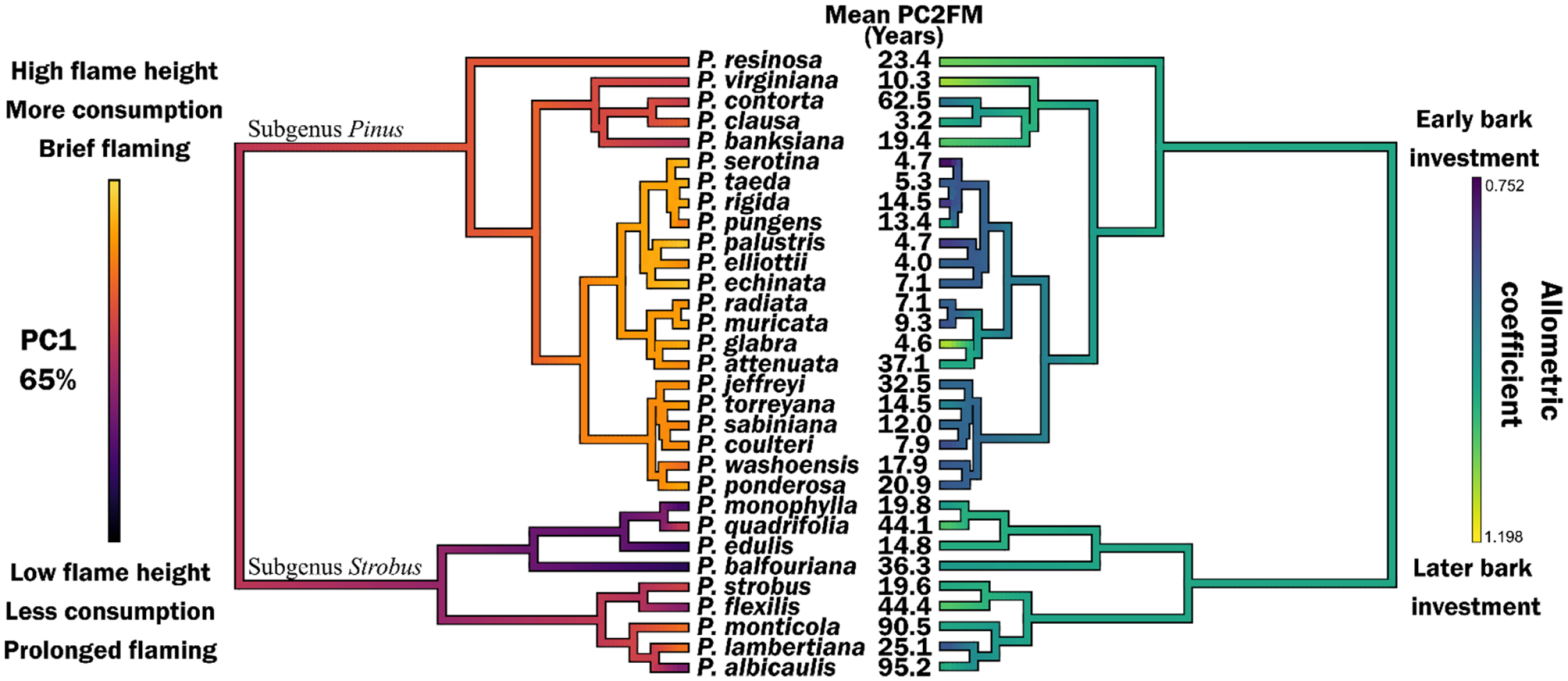
Phylogeny of 31 *Pinus* species in North America examined in this study. The left tree is colored to indicate litter flammability based on the first principal component axis. The right tree is colored based on the allometry of fire-protective bark developed by^7^.

**Figure 5:**
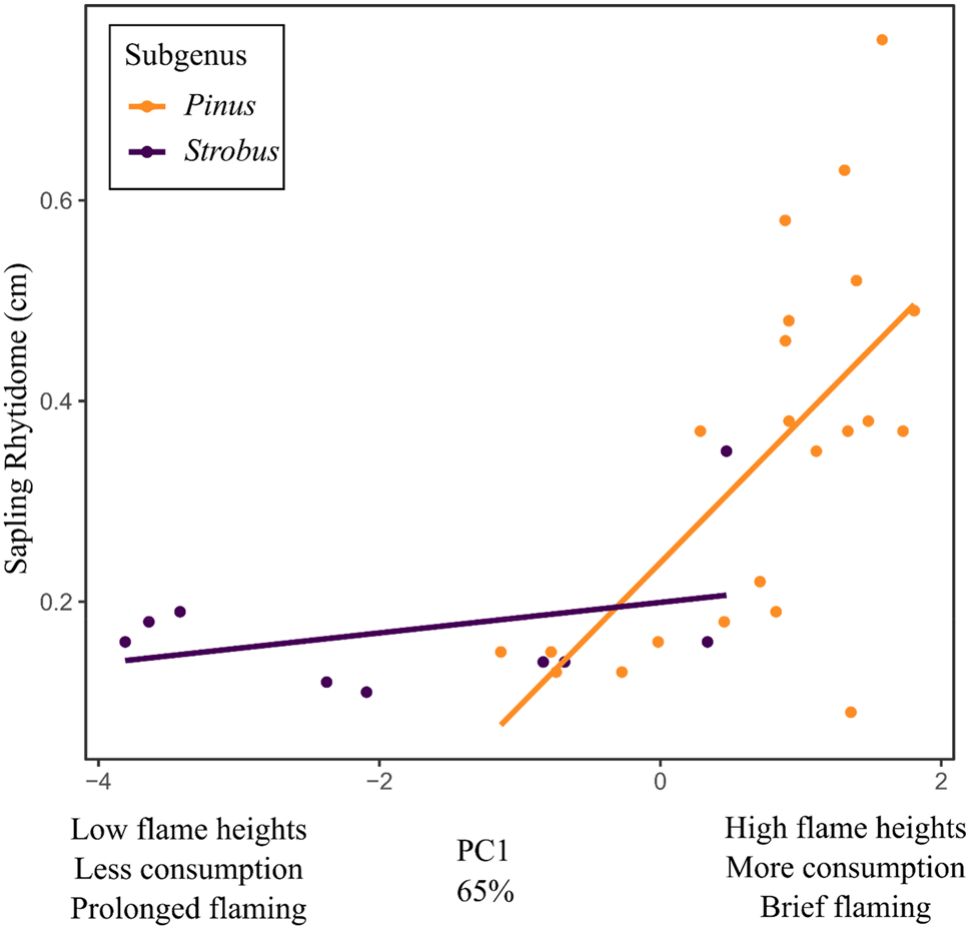
Relationships between litter flammability (based on the first principal component of flammability metrics) and sapling rhytidome thickness (based on allometric equations for a 5 cm diameter stem^7^) for 31 North American pine species.

Patterns in bark investment were also variable among the pines and within the two subgenera. Subgenus *Pinus* was somewhat variable; most species had early investment in bark although there were notable species that contradicted this (*P. resinosa*, *P. virginiana*, *P. banksiana*, *P. glabra*, *P. clausa*, and *P. attenuata*; Fig. 4). All of the *Strobus* subgenus, except *P. lambertiana*, had late bark investment. The Australes subsection of the phylogeny (subgenus *Pinus*) was distinct for having both early investment in bark as well as high litter flammability (Fig. 4). Mean sapling rhytidome based on the allometric equations^7^ was significantly thicker in the *Pinus* subgenus than in the *Strobus* subgenus (0.34 and 0.17 cm respectively, *t* = 3.677, *df* = 28.981, *p* = 0.001). The PGLS model of sapling rhytidome thickness was associated (adjusted *R*^2^ = 0.46, *p* = 0.0001) with flammability (*p* = 0.0001) and its interaction with subgenus (*p* = 0.007, Fig. 5). Fitting models to both subgenera separately revealed that flammability was correlated with bark thickness for the *Pinus* subgenus (adjusted *R*^2^ = 0.39, *p* = 0.001) but not for the *Strobus* subgenus (adjusted *R*^2^ = 0.001, *p* = 0.35).

### Linking fire regime to fire-adapted traits

**Figure 6:**
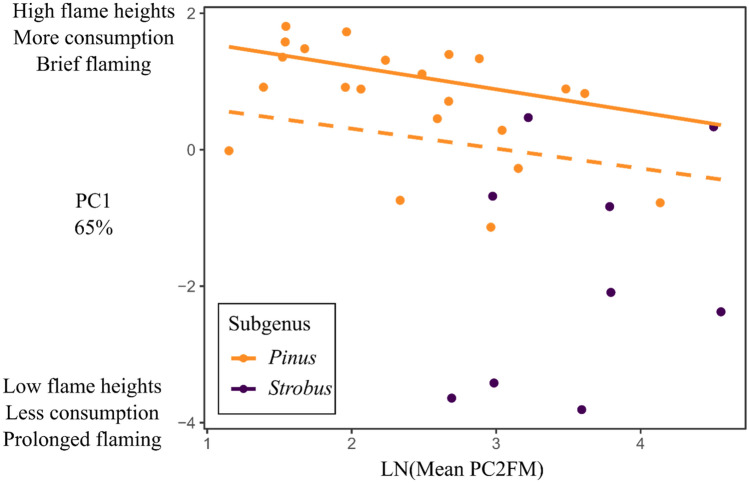
Relationship between flammability (PC1) and log transformed mean fire return interval based on PC2FM for species in the *Pinus* and *Strobus* subgenera. Dashed line represents PGLS regression line for the entire *Pinus* subgenus. Solid line represents the PGLS regression without the five species in the *Pinus* and *Contortae* subsections. No significant model could be fit to the *Strobus* subgenus.

The PC2FM mean fire return interval prior to 1850 was significantly lower for species in the *Pinus* subgenus with mean estimated fire return intervals of 15.3 and 43.3 years for species in the *Pinus* and *Strobus* subgenera respectively (*t* = −2.6799, *df* = 9.4485, *p* = 0.02). The PGLS of fire return interval was not significant for the entire dataset even when subgenus was included as an interaction (*R*^2^ = 0.04, *p* = 0.26, λ = 0.71, 95% CI = 0–0.96). A negative correlation between fire return interval and PCA1 was found when fitting the model to the *Pinus* subgenus with a significant phylogenetic signal (*R*^*2*^ = 0.16, *p* = 0.04, λ = 0.84, 95% CI = 0.49–0.99). Plotting the relationship (Fig. 6) revealed that five species in the *Pinus* subgenus seem to be outliers for the trend. These species belonged to the *Pinus* (*P. resinosa*) and *Contortae* (*P. banksiana*, *P. clausa*, *P. contorta*, and *P. virginiana*) subsections of the genus. Removing these species slightly improved the model and removed the phylogenetic signal (*R*^2^ = 0.24, *p* = 0.025, λ = 0, 95% CI = 0–0.46).

## Discussion

Flammability of North American pine litter varies widely across the genus from species with highly flammable, fast burning litter that was more readily consumed to species with litter that burned with low flames, for extended duration, and with less consumption. Similar variability in flammability among eight eastern and western USA pine species was found and attributed the variation to different fire adaptive strategies^19^. Variability in litter flammability has been seen in other genera as well. For example, oak (*Quercus* spp.) litter flammability has been found to vary similarly, with species that occur in fire-prone areas generally having litter that is more flammable^37,38^. This pattern is also seen across species of different genera, for example in western conifers of the genera *Abies* and *Picea*^18,35^.

The pine litter flammability experiments in this study revealed a link between the distribution of pine species and the historical mean fire return interval estimates of^39^. Guyette et al.’s^39^ mapped PC2FM estimates (Fig. 4 in^39^) used temperature, precipitation, and partial pressure of oxygen to estimate the frequency of fire from 1650 to 1850 CE. Our study, for example, showed that *P. palustris* litter had high flame heights, high consumption, and short flame duration (Appendix 2). The species distribution of *P. palustris* also coincides with an historical mean fire return interval of < 2 to 4 years in^39^. Dendrochronology studies on fire scarred trees have confirmed frequent fires occurring every 2.2 years on average in these ecosystems^40^. This pattern of flammability matching fire regimes also holds for the other pines with high flammability (the eastern *Australes* subsection including *P. echinata*, *P. serotina*, *P. taeda*, and *P. rigida* plus the western *P. sabiniana*, *P. washoensis*, and *P. muricata*). The PGLS model was slightly better when fit without the *Pinus* and *Contortae* subsections, however this could be due to a lack of representation in these groups. The lack of phylogenetic signal when removing these subsections may be an artifact of lower sample size which increases the likelihood of Type II error in PGLS^41^. In contrast, the *Strobus* subgenus showed no relationship between flammability and fire return interval, likely due to these species not having a strong selective pressure from frequent fire. For example, *P. balfouriana* had low flame heights, minimal consumption, and long flame time in our burning experiments. The native range of *P. balfouriana* has a mean fire return interval of 50 + years in PC2FM. The other pines with low flammability (*P. edulis*, *P. monophyla*, *P. flexilis*, and *P. albicualis*; all members of subgenus *Strobus*) track this pattern: arid or montane pines in fire-infrequent fire regimes in the western USA. Fires occurring so infrequently allow the pine species to reach sexual maturity before fires occur on average, minimizing the selective pressure that fire exerts in frequent fire regimes.

Our results suggest that litter flammability of species in the *Strobus* subgenus was influenced more by physical traits, specifically needle length, than species in the *Pinus* subgenus. Needle length explained more of the variability in the *Strobus* subgenus and the slope of the regression was steeper. Physical leaf traits have been found to explain significant portions of the variability in litter flammability in other studies. Physical leaf traits (length, width, perimeter, and curling height) were significant drivers of litter flammability in Californian *Quercus* species and their allies^38^. Flammability of mixed species fuelbeds in the Sierra Nevada were also driven by the abundance of longer-leaved pine species^42^.

In contrast to previous links between flammability and litter traits, substantial variation within *Pinus* was unexplained by these traits alone, particularly in the *Pinus* subgenus. We hypothesize that more of the variation in flammability in the *Pinus* subgenus may be explained by variations in chemical traits of the litter, specifically terpene concentrations. For example, species of *Pinus* and *Cistus* with higher litter terpene content burned with taller flame heights, more rapid spread rates, and shorter combustion times^43^. Terpene concentrations are strongly linked to the phylogeny of the pine genus (e.g.^44,45^), and are useful genetic markers in studies of evolution and systematics because they are not influenced by environmental conditions^46,47^. We found a significant phylogenetic signal in the residuals of the flammability model for the *Pinus* subgenus which may be accounted for by the relationship between terpenes and phylogeny. The lack of a significant phylogenetic relationship in the *Strobus* subgenus should be taken with caution however, because the low number of species in our collections (*n* = 9) likely inflated the high type II error rate^41^. Expanding collection of other *Strobus* species, particularly in the species-rich Mexican flora, offers an opportunity to clarify this potential relationship. Future studies on pine flammability should consider chemical composition of the litter in addition to phylogenetic relationships and physical traits.

Our study follows on recent attempts to relate litter flammability to other fire adaptive traits (here, bark thickness). High fire resistance traits in western conifers (thick bark, self-pruning, and flammable litter) closely agreed with their historical fire regimes^35^. In our study, the significant correlation between litter flammability and sapling bark thickness in the *Pinus* subgenus but not in the *Strobus* subgenus is consistent with the hypothesis of^48^ that fire beginning in the Cretaceous period influenced trait evolution in *Pinus*. The split of the genus into *Pinus* and *Strobus* subgenera is widely believed to have occurred sometime in the Cretaceous^2,48,49^. The two subgenera diverged, likely as a result of competition with angiosperms, to inhabit different environmental conditions^2^. The *Strobus* subgenus largely adapted toward stressful conditions such as alpine and desert environments, while the *Pinus* subgenus took advantage of fire-disturbed environments^2,4^. The stressful habitats where most species in the *Strobus* subgenus are found rarely experience fire at regular intervals and therefore the selective pressure to develop thick bark at young ages was infrequently experienced.

Our understanding of drivers of flammability are evolving. In contrast to *Quercus*, where clades failed to explain much variation in flammability^38^, pine traits follow phylogenetic differences more closely. It may be that in many species (as in the subgenus *Pinus* here), physical traits drive flammability. In others (as in subgenus *Strobus*), combinations of physical and chemical traits may drive the process. Aside from establishing that species differ (as many in this field have found and reported), a better understanding of the underlying drivers of differential flammability remains a major thrust of determining the role of historic fire regimes on species trait evolution and how traits determine dominance under future fire regimes.

## Methods

We collected litter from 31 pine species from their native ranges (Fig. 1; SI: Appendix 1). Species were collected from wild forest, woodland, and savanna populations across the US, from Massachusetts to California and Wisconsin to Florida. For all species, recently senesced foliar litter was collected from the surface of the superficial Oi (litter) horizon soon after leaf fall. For each species we collected approximately 20 g of litter from beneath 5 to 10 individual trees across the site. Samples were stored in paper bags and transported to the laboratory where they air dried. All pine litter collections were made with approvals from relevant agencies or landowners, where required.

**Figure 7:**
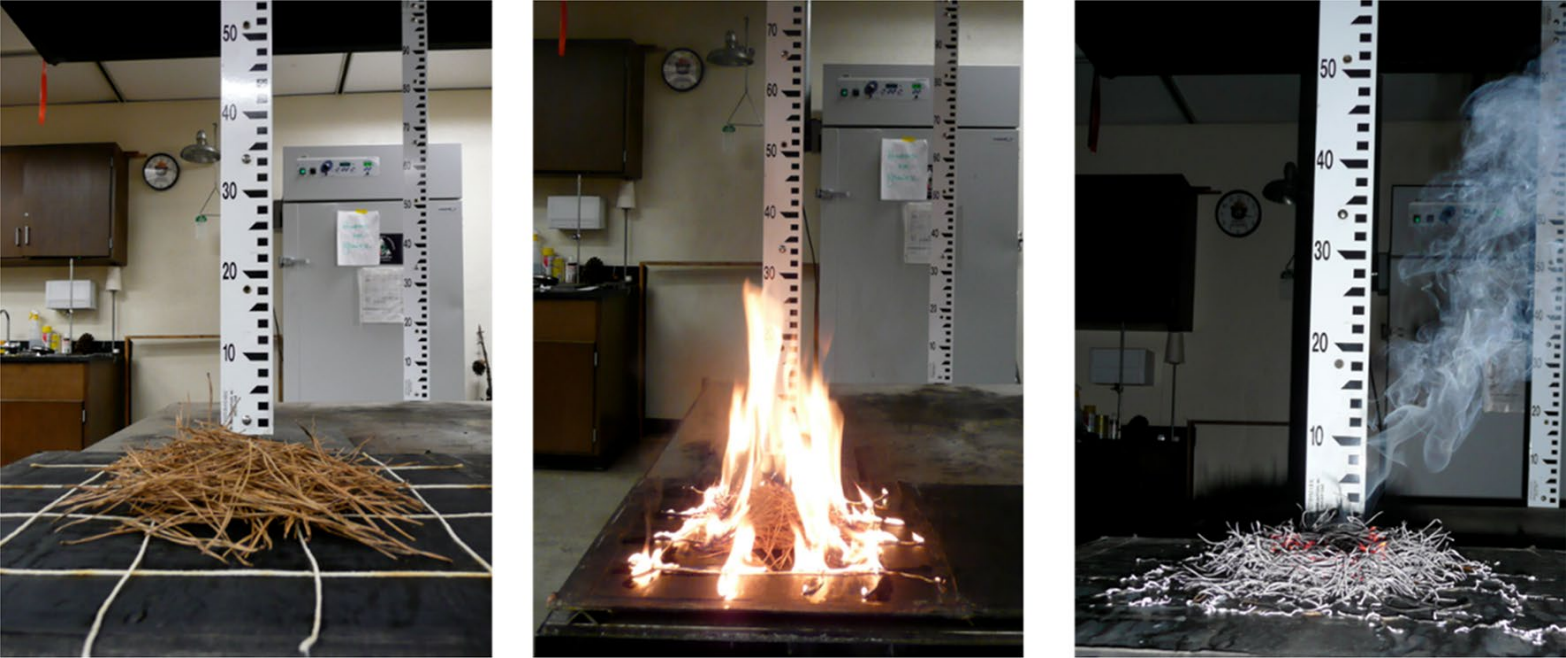
An example of pine litter flammability experiment (here, *Pinus glabra*) from pre-ignition (left), during flaming (middle), and smoldering (right).

In the laboratory, we measured species-level traits, including needle length (cm; with a ruler) and needle thickness (mm at leaf midpoint; with digital calipers) of subsamples from each species. Next, all litter samples were oven-dried at 60 °C for 24 h; we used this low temperature heating to minimize loss of volatiles that may be important in flammability. Surface litter temperatures in the field typically exceed 60 °C in open-canopied sites^50^, so we felt confident that our drying treatments minimized artificial volatile loss. Dried litter was weighed [target mass was 15.0 g; range was 14.97 g to 15.16 g (SE = 0.02 g)] distributed over a 4 × 4 lattice of cotton string infused with xylene within a 25 cm × 25 cm area on top of a stainless steel platform, consistent with other published flammability experiments^19,22,37^. Once the oven-dried litterbeds were created, we took four litter depth measurements 7 cm diagonally from each corner of the litterbed and calculated the average depth for each sample (Fig. 7). Our methodology relied on “reconstructed” litter beds (as in^51^); we acknowledge those limitations, but using the same conditions, mass, and arrangements allowed us to compare species rather than what are typically variable conditions for each species in their relevant field settings.

All burning experiments were conducted beneath a 2 m × 3 m fume hood under controlled laboratory conditions (as in^19,37^ and others). Draw generated by a fan within the exhaust chimney, measured at the hood and chimney interface, was approximately 15–20 cm s^−1^; however, no detectable air movement was measured above the litterbed. We ignited the xylene-soaked strings beneath the litter fuels in rapid succession. Once the pine litter sample ignited, two trained observers measured the duration (sec) and maximum height (cm) of the flames. Once flaming ceased, the duration of smoldering (glowing; sec) combustion was measured until extinction (judged with turning off overhead lighting to ensure no glowing combustion). After extinction, all unburned string was removed from the burned sample and the ash and unburned litter were weighed to calculate fuel consumption (% of original mass). All litter beds ignited for all species and replicates.

Mean flame duration, flame height, smoldering duration, and percent consumption for each species were analyzed with principal components analysis (PCA) using the “prcomp” function in the R package *stats*^52^. The PCA reduces the dimensionality of the data and removes correlations among flammability metrics^38^. The principal component axis that explained the largest amount of variance in the data (PC1) was used in subsequent analyses to compare flammability with physical traits.

Comparisons among species can violate the assumption that points are independently drawn from a common distribution because closely related species would have similar evolutionary histories^53^. To account for this, we conducted phylogenetic generalized least squares regression analyses (PGLS) using the *pgls* function in the R package *caper* to examine the relationships between physical traits and flammability^54^. In each model, Pagel’s λ^55^ was first estimated using the maximum likelihood method in^56^. Pagel’s λ typically varies from 0 (no phylogenetic correlation) to 1 (traits covary proportionately to their evolutionary history) and incorporates the phylogenetic relationships into the estimated covariance of the residuals as needed assuming a Brownian motion of evolution^56^. The adjusted *R*^2^ values reported here show the proportion of variance explained given the same phylogenetic covariance matrix between the null model and the actual model^54^. Our analyses used the pine phylogeny presented by^49^, which was developed using the fossilized birth–death method on 21 pine fossils. We fit multiple PGLS models to test whether physical traits and phylogeny explain variation in flammability, using PC1 as the response variable and the physical traits (needle length, thickness, and litter depth) and subgenus as predictor variables. Twelve models were compared, testing physical traits alone (both untransformed and log transformed) as well as interactions with subgenus, using the *model.sel* function in the R package *MuMIn*^57^.

We also used PGLS to examine correlations between flammability and bark thickness. For these models, sapling bark thickness (the most vulnerable growth stage to frequent fire) was used as a response variable and PC1 was used as the predictor. Outer bark thickness was estimated for each species using the allometric equations developed by^7^ for a sapling with a diameter of 5.0 cm. To evaluate the correlation differed between the two lineages in the *Pinus* genus, we fit separate models to the *Pinus* and *Strobus* subgenera.

To assess whether historic fire return interval was correlated with flammability, we used the results of^39^ PC2FM (Physical Chemistry Fire Frequency Model) estimates of fire intervals prior to 1850. We calculated the mean fire return interval for each species using PC2FM for each species’ natural range^58^. We compared mean fire return intervals and sapling rhytidome thickness between the two pine subgenera using *t*-tests as well as using PGLS to examine the correlation between PCA1 and log transformed mean fire return interval. We again fit separate models to the two pine subgenera as well as subsets of the *Pinus* subgenus when outliers were present.

## Supplementary Information

Supplementary Tables.

## Data Availability

Mean flammability trait data for all pine species are provided in the Supplemental Information; Appendix 2. All flammability, bark allocation, and fire return interval data will be deposited in Dryad at publication.
