# Supplementary material for: Understanding flammability and bark thickness in the genus Pinus using a phylogenetic approach
Source: Sci Rep. 2022 May 5;12:7384. doi: 10.1038/s41598-022-11451-x (PMC9072376; doi:10.1038/s41598-022-11451-x)
Supplement: Supplementary file 1 — Supplementary Tables. [file 41598_2022_11451_MOESM1_ESM.docx]

| SI: Appendix 1. *Pinus* species and litter collection locations. | | | | |
| --- | --- | --- | --- | --- |
| Species | Common name | Subgenus | Location |  |
| *Pinus albicaulis* | whitebark pine | Strobus | Mount Rose, NV |  |
| *Pinus attenuata* | knobcone pine | Pinus | King Range, CA |  |
| *Pinus balfouriana* | foxtail pine | Strobus | Trinity Alps, CA |  |
| *Pinus banksiana* | jack pine | Pinus | Cas County, WI |  |
| *Pinus clausa* var. *clausa* | Ocala sand pine | Pinus | Ocala National Forest, FL |  |
| *Pinus contorta* subspecies *bolanderi^†^* | Bolander pine | Pinus | Mendocino County, CA |  |
| *Pinus contorta* subspecies *contorta^†^* | shore pine | Pinus | Patrick’s Point, CA |  |
| *Pinus contorta* subspecies *murrayana^†^* | Sierra lodgepole pine | Pinus | Lake Tahoe, NV |  |
| *Pinus coulteri* | Coulter pine | Strobus | Cleveland NF, CA |  |
| *Pinus echinata* | shortleaf pine | Pinus | Shelby County, AL |  |
| *Pinus edulis* | pinyon pine | Pinus | San Francisco Peaks, AZ |  |
| *Pinus elliottii* var. *elliottii* | slash pine | Pinus | Baker County, GA |  |
| *Pinus flexilis* | limber pine | Strobus | White Mountains, CA |  |
| *Pinus glabra* | spruce pine | Pinus | Apalachicola Ravines, FL |  |
| *Pinus jeffreyi* | Jeffrey pine | Pinus | Lake Tahoe, CA |  |
| *Pinus lambertiana* | sugar pine | Strobus | Lake Tahoe, CA |  |
| *Pinus longaeva* | Gr. Basin bristlecone pine | Strobus | White Mountains, CA |  |
| *Pinus monophyla* | single-leaved pinyon pine | Pinus | White Mountains, CA |  |
| *Pinus monticola* | western white pine | Strobus | Horse Mountain, CA |  |
| *Pinus muricata* | Bishop pine | Pinus | Patrick’s Point State Park, CA |  |
| *Pinus palustris* | longleaf pine | Pinus | Baker County, GA |  |
| *Pinus ponderosa* | ponderosa pine | Pinus | Mendocino County, CA |  |
| *Pinus pungens* | Table Mountain pine | Pinus | Table Mountain, SC |  |
| *Pinus quadrifolia* | four-leaved pinyon | Pinus | San Diego County, CA |  |
| *Pinus radiata* | Monterey pine | Pinus | Monterey County, CA |  |
| *Pinus resinosa* | red pine | Pinus | Oneida County, WI |  |
| *Pinus rigida* | pitch pine | Pinus | Montague Plain, MA |  |
| *Pinus sabiniana* | ghost pine | Pinus | Trinity County, CA |  |
| *Pinus serotina* | pond pine | Pinus | Ocala National Forest, FL |  |
| *Pinus strobiformis* | Southwestern white pine | Strobus | San Francisco Peaks, AZ |  |
| *Pinus strobus* | eastern white pine | Strobus | Vilas County, WI |  |
| *Pinus taeda* | loblolly pine | Pinus | Tuskegee National Forest, AL |  |
| *Pinus torreyana* | Torrey pine | Strobus | Torrey Pines State Park, CA |  |
| *Pinus virginiana* | Virginia pine | Pinus | Shelby County, AL |  |
| *Pinus washoensis* | Washoe pine | Pinus | Galena Creek Co. Park, NV |  |

*^†^* only *Pinus contorta* subspecies *contorta* was used in the analyses.

| SI: Appendix 2. Laboratory flammability metrics of *Pinus* litter. | | | | |
| --- | --- | --- | --- | --- |
| Species (*n*) | Flame height (cm) | Flame duration (sec) | Smoldering duration (sec) | Fuel consumption (%) |
| *Pinus albicaulis* (7) | 48.4 | 182.9 | 498.1 | 63.4 |
| *Pinus attenuata* (7) | 79.9 | 53.4 | 635.6 | 89.5 |
| *Pinus balfouriana* (7) | 20.4 | 114.3 | 405.0 | 30.1 |
| *Pinus banksiana* (5) | 49.4 | 149.4 | 258.8 | 71.5 |
| *Pinus clausa* var. *clausa* (7) | 65.0 | 81.7 | 488.0 | 80.8 |
| *Pinus contorta* subsp. *bolanderi* (5) | 59.6 | 54.9 | 584.0 | 77.5 |
| *Pinus contorta* subsp. *contorta* (7) *^†^* | 48.0 | 93.4 | 497.2 | 79.6 |
| *Pinus contorta* subsp. *murrayana* (7) | 66.9 | 155.3 | 444.4 | 82.5 |
| *Pinus coulteri* (7) | 67.7 | 82.4 | 328.6 | 92.0 |
| *Pinus echinata* (7) | 84.9 | 42.7 | 272.4 | 85.6 |
| *Pinus edulis* (7) | 40.0 | 253.9 | 588.3 | 62.2 |
| *Pinus elliottii* var. *elliottii* (7) | 70.9 | 81.7 | 346.3 | 90.9 |
| *Pinus flexilis* (7) | 55.3 | 154.6 | 769.4 | 71.7 |
| *Pinus glabra* (7) | 78.3 | 53.1 | 331.9 | 87.6 |
| *Pinus jeffreyi* (7) | 83.9 | 105.1 | 410.7 | 89.0 |
| *Pinus lambertiana* (7) | 68.9 | 74.6 | 513.9 | 88.8 |
| *Pinus longaeva* (7) | 25.7 | 239.3 | 529.4 | 44.6 |
| *Pinus monophyla* (7) | 28.1 | 149.7 | 801.7 | 62.4 |
| *Pinus monticola* (7) | 75.1 | 90.3 | 481.7 | 82.8 |
| *Pinus muricata* (4) | 84.3 | 66.5 | 483.3 | 92.9 |
| *Pinus palustris* (6) | 86.8 | 57.0 | 330.7 | 92.4 |
| *Pinus ponderosa* (7) | 64.9 | 111.1 | 363.3 | 88.8 |
| *Pinus pungens* (7) | 69.0 | 74.0 | 476.9 | 86.3 |
| *Pinus quadrifolia* (7) | 54.7 | 64.7 | 701.9 | 76.5 |
| *Pinus radiata* (7) | 72.0 | 38.6 | 595.6 | 92.2 |
| *Pinus resinosa* (5) | 65.0 | 140.2 | 476.2 | 88.4 |
| *Pinus rigida* (6) | 85.5 | 89.0 | 242.0 | 87.0 |
| *Pinus sabiniana* (7) | 76.1 | 75.7 | 327.9 | 88.8 |
| *Pinus serotina* (10) | 87.2 | 89.1 | 213.2 | 88.5 |
| *Pinus strobiformis* (7) | 69.7 | 62.1 | 740.4 | 81.2 |
| *Pinus strobus* (5) | 64.2 | 158.0 | 287.2 | 74.0 |
| *Pinus taeda* (7) | 84.1 | 72.0 | 261.1 | 86.9 |
| *Pinus torreyana* (8) | 66.3 | 65.9 | 397.4 | 88.4 |
| *Pinus virginiana* (7) | 51.4 | 92.9 | 512.4 | 78.4 |
| *Pinus washoensis* (7) | 84.9 | 57.7 | 495.0 | 91.4 |

*^†^* only *Pinus contorta* subspecies *contorta* was used in the analyses.
